# Long-term learning and forgetting of feature binding in verbal free recall

**DOI:** 10.1177/17470218221111343

**Published:** 2022-07-13

**Authors:** Riccardo Sacripante, Sergio Della Sala, Robert H Logie

**Affiliations:** Human Cognitive Neuroscience, Department of Psychology, University of Edinburgh, Edinburgh, UK

**Keywords:** Feature binding, visual short-term memory, long-term learning, forgetting

## Abstract

Temporary feature bindings can be learned under specific experimental conditions. However, how this learning occurs and how it is forgotten over long intervals is unclear. We addressed this question with repeated presentation of an array of coloured shapes followed by verbal free recall after delays of 1 day, 1 week, and 1 month. A total of 120 participants viewed 24 repetitions of the same study array of six objects each with two features (shape and colour). After 24 trials, 61 participants reported becoming aware of the repetition while 59 reported being unaware. Memory performance improved across trials, with aware participants showing faster learning than unaware participants whose performance appeared to reflect the capacity of short-term visual memory across all repetitions. Both aware and unaware participants recalled some of the array after their allocated delay, showing that learning had occurred during repetition trials, even for unaware participants who showed little or no improvement across 24 repetition trials. Memory for binding showed no change after 1 day compared with performance on the 24th repetition trial, was significantly lower for participants tested after 1 week, and was lower still for those tested after 1 month. Findings are interpreted as consistent with both a short-term, limited capacity visual cache that supports performance during early repetition trials, before learning can have occurred, and gradual strengthening across trials of an episodic long-term memory trace that supports learning. If the episodic trace exceeds the threshold of awareness, this accelerates learning.

## Introduction

Integrating different visual features into complex, detailed information is a cognitive function that we use in day-to-day life, known as binding. Studies of feature binding have predominantly focused on temporary memory, leaving open the question of whether, and under what circumstances, such bindings lead to long-term learning. Even when a feature binding is held only briefly for the current task, is there, in addition, the formation of an episodic trace of specific bindings that can later be retrieved? For example, while driving it is important on a moment-to-moment basis to update our memory for the current colour of a traffic light and of the changing position of vehicles around us. However, if a specific traffic light spends a long time on red every time we drive to that junction, then we might learn to avoid that junction and take an alternative route. In an experiment, on one trial, we might see a red circle and a blue square, but on a later trial the array might contain a blue circle and a red square, and memory for the previous array would hamper memory performance on the current and subsequent trials. So, there is an advantage to forgetting a specific colour–shape combination as soon as a trial is completed. While there are potential disadvantages to retaining feature bindings trial to trial in an experiment, and in rapidly changing environments in everyday life, there is the possibility that both visual short-term memory or a temporary, limited capacity “visual cache” (Logie, 1995, 2003, 2016; Logie et al., 2021) and episodic long-term memory might be recruited. Yet only a handful of studies has examined this possibility by investigating whether there is learning of repeated visual feature combinations (Colzato et al., 2006; Logie et al., 2009; Shimi & Logie, 2019).

Dishon-Berkovits and Treisman (reported in Treisman, 2006) carried out a preliminary experiment to investigate the interaction between visual short-term memory and long-term memory with a change detection paradigm using a single probed item. In this experiment, some combinations of shapes and colours were repeated in 80% of the trials and their long-term learning and explicit recall was assessed through a surprise post-experiment questionnaire. While participants seemed to perform better than chance on the post-experiment questionnaire, their performance on the visual short-term memory task did not seem to benefit from such repetition. This was in contrast with a range of previous studies that have shown clear and rapid learning of repeated verbal and non-verbal sequences (e.g., Couture & Tremblay, 2006; Cunningham et al., 1984; Fastame et al., 2005; Hebb, 1961; Page et al., 2006; Szmalec et al., 2009). However, in those previous studies, the focus was on recall of verbal or visual serial order of a repeated sequence, not of a single array of multiple items. Dishon-Berkovits and Treisman concluded that, in their task involving change detection, visual working memory supported performance on each trial, although there was in addition gradual learning of the array. Long-term memory did not contribute to performance during the repetition trials, but its influence could be detected post-experiment. That is, visual working memory and long-term memory acted as separate memory stores for colour–shape bindings. Colzato et al. (2006) reported a similar dissociation and concluded that the ability to temporarily bind features and to learn their combinations may rely on independent mechanisms.

Using a probed recall procedure with verbal recall of the names of bound colours and shapes as employed in previous research (Brockmole et al., 2008; Gajewski & Brockmole, 2006), Logie et al. (2009, Experiment 3) observed substantial learning of repeatedly presented arrays of six integrated objects in a cued recall task. However, in striking contrast, with a change detection paradigm, Logie et al. (2009, Experiments 1 and 2) observed no evidence for learning following 60 repetitions of the same visual array. Hence, learning appeared to arise from repeated recall of the stimulus array, not from repeated presentation, and performance in change detection was thought to be supported by a temporary, limited capacity visual buffer, referred to as the visual cache (Logie, 1995), not by episodic long-term memory. It was assumed that the contents of the visual cache were overwritten by the stimulus on the following trial, even if the stimulus was identical across trials. Therefore, there was no residual trace from trial to trial within the visual cache that could support the accumulation of learning. This was consistent with the Dishon-Berkovits and Treisman, and the Colzato et al. argument that temporary binding and learning rely on separate cognitive functions.

Shimi and Logie (2019) further explored the relationship between the short-term retention and long-term learning of feature binding, contrasting a change detection task (Experiment 1) with a visual reconstruction task (Experiment 2), and with 120 repetitions of the same stimulus array, double the number of repetitions used by Logie et al. (2009). Reconstruction involved participants viewing at test, the full array of colours, of shapes, and of locations displayed separately on the screen, and selecting the colour, shape, and location for each item shown in the study array. For six-item arrays, reconstruction resulted in rapid learning of the repeated feature bindings, with performance reaching asymptote close to ceiling after 60 of the 120 trials. In contrast, the change detection task with six-item arrays resulted in very slow learning, and participants failed to reach ceiling after 120 trials. Participants were also asked at the end of the experiment whether they had been aware of the repetition. Participants who reported becoming aware showed faster learning than those who reported not being aware, with participants in the latter group showing very little improvement in performance, even with 120 repetitions of the same array. In an additional experimental condition, participants were shown four-item arrays repeated for 120 trials. In this case, all participants were close to ceiling on trial 1, when no learning could have occurred, and maintained this level for all 120 trials.

The Shimi and Logie (2019) results were interpreted as consistent with the Logie et al. (2009) conclusion that rapid learning occurs as a result of repeatedly recalling, or in the later study, reconstructing the same stimulus array across trials, and learning is very much slower when based solely on recognition of a repeated stimulus array as occurs with change detection. On the first few trials, before any learning can occur, for both reconstruction and change detection, performance is thought to be supported by a limited capacity visual cache. The finding that four-item arrays resulted in close to ceiling performance on trial 1 suggests that this size of array reflects the capacity of the visual cache. For six-item arrays, performance on trial 1 was around 70%, equivalent to remembering four items, so again consistent with reliance on the capacity of the visual cache. Repeated reconstruction with six-item arrays was assumed to result in a rapidly strengthening episodic trace that then increasingly supported performance across trials, with decreasing reliance on the visual cache. For change detection, again, for the first few trials, no learning could have occurred, and so performance could rely only on a temporary representation, interpreted as the visual cache. Across change detection trials, Shimi and Logie (2019) argued that there was a weak episodic trace formed initially that gradually strengthened, and it was only after many trials that the trace became sufficiently strong to support performance, with continuing support also from the visual cache. If the build-up of that episodic trace led to the participant becoming aware of the repetitions, that allowed faster strengthening of the episodic trace, thereby accelerating learning. In sum, the authors concluded that learning in the reconstruction task was enhanced by the support of both a temporary visual cache on each individual trial and learning in episodic long-term memory of the repeated reconstruction (rather than repeated presentation) of the same array. In change detection, performance was thought to be supported by the visual cache for each individual trial, and by the formation of a weak episodic trace that required many repetition trials for learning to accumulate and provide additional support for performance.

Overall, this line of research has focused primarily on short-term retention of feature bindings and has shown the experimental conditions under which feature combinations can be learned, albeit slowly in change detection, and more rapidly under experimental conditions that require visuo-motor reconstruction or cued verbal recall of the items.

However, it is an open question whether participants who show little or no improvement across repetitions are continuing to rely on a short-term visual representation on each trial, or if they are indeed learning, but only for a subset of items in the array. That is, they do not learn the entire array, but do learn perhaps four out of the six items presented, so there is little or no improvement in performance across repeated trials. One way to test this is to consider whether participants can recall items from the repeated array at various longer intervals after the initial experimental session. If recall is above chance after an extended delay, it would provide clear evidence of learning, even if this evidence was lacking during the initial test session. Of particular interest is whether participants who show very little learning (anticipated to be those reporting that they were not aware of the repetition) can perform above chance after an extended delay. This would provide evidence that they have learned at least part of the array, even if that learning has not resulted in an improvement in performance across the initial repeated trials.

Although the focus of previous papers on this topic has been on memory for visual feature binding, it is notable that several studies have used a verbal response to test visual short-term memory, either in response to a spatial cue (e.g., Brockmole et al., 2008; Gajewski & Brockmole, 2006; Guazzo et al., 2020; Logie et al., 2009, Experiment 3), or with verbal free recall of the names of colours and shapes (Della Sala et al., 2012; Killin et al., 2018; Parra et al., 2009, 2015). It is with repeated verbal recall (or repeated visual reconstruction) that learning effects have been most evident in those previous studies, and so we adopted verbal free recall of visual arrays of bound features (colours and shapes) to maximise the possibility that we would observe learning, thereby allowing for an investigation of long-term forgetting.

The present study aimed to assess the extent to which learning has occurred as a result of repeated verbal recall of the same visual array by testing memory after 1 day, 1 week, and 1 month. To avoid relearning from repeated testing, different groups of participants were tested after each time interval.

Logie et al. (2009, Experiment 3) found that cued verbal recall led to rapid learning within a first block of 20 trials. In the present study, we repeated the same 6 colour–shape combinations across 24 trials and tested memory with verbal free recall of the colour and shape names for each object after each presentation. This was intended to maximise the chances that participants would learn across repetitions. At the end of the 24 repeated trials, participants were asked if they became aware that the same colour–shape combinations had been repeated during the test session. We expected that higher levels of performance would be observed for participants who reported becoming aware of the repetition compared with those reporting that they were unaware, and that recall performance would be above chance for both aware and unaware groups, and for each delay group.

## Methods

### Participants

A group of 120 young adults (79 women and 41 men) aged between 18 and 35 years (*M* = 25.2, *SD* = 4.01) with normal or corrected-to-normal vision and normal colour vision were recruited to take part in the experiment. Their years of education ranged from 12 to 18 years (*M* = 16.82, *SD* = 1.52). All participants were fluent English speakers.

Statistical power for the minimum required sample size was determined a priori by performing a power analysis with G*Power 3 (Faul et al., 2007). Following Shimi and Logie (2019), the power analysis was based on a $\eta_{p}^{2}$ of .06 (medium size effect) with the aim to achieve a power of 0.99 at a significance level of α = .05. The estimated number was 38 participants for each group, respectively, for testing after 1 day, 1 week, and 1 month. According to this, 40 participants were assigned to each group.

Participants were given a small honorarium and were asked to sign a consent form before formally starting the experiment. Ethical approval for this study was provided by the Psychology Research Ethics Committee of the University of Edinburgh.

Half of the participants (*n* = 60) were initially tested individually in a laboratory at the Department of Psychology of the University of Edinburgh; however, due to the COVID-19 pandemic (World Health Organization, 2020), the remaining participants (*n* = 60) were tested online, individually via live video link (see Table 1).

**Table 1.** Sample sizes for each analysis reported.

Analysis	*n* (total)	Groups	Notes
1	120	Onsite/Online (learning)	60 tested onsite versus 60 tested online
2	120	Aware/Unaware (learning)	61 aware versus 59 unaware
3	118	Aware/Unaware + Delay (forgetting)	18 aware versus 22 unaware participants (1 day group), 19 aware versus 21 unaware (1 week), 22 aware and 16 unaware (1 month)
4	118	Delay groups (trial 1/follow-up)	40 after 1 day, 40 after 1 week, 38 after 1 month

### Material

The task used in this experiment was based on that used by Shimi and Logie (2019), Experiment 1. The stimulus consisted of an array of six items arranged symmetrically around an imaginary circle, each at a 5.63° from a central fixation point. The visual array was created by combining six colours (blue, green, red, white, yellow, and grey) each with six geometric shapes (square, cross, triangle, heart, circle, and star) without replacement. The stimulus was presented on a black background using E-Prime 2.0 (E-Studio, Psychology Software Tools Inc.).

Two different test arrays were generated by varying the location and the colours of the shapes, and each participant was presented with one or other variation of the array. However, the precise combinations of colours and shapes in the visual array were kept constant for any one participant throughout the learning session.

### Experimental procedure

Participants attended an initial session, either in our lab or via a live video session during which they were seated in front of a computer at an approximate distance of 50 cm, with instructions and monitoring of participation by the experimenter in person or through the live video link. Instructions about the experiment were given orally and in written format on screen. The session took approximately 20 min overall.

At the beginning of the task, participants were given three random practice arrays that were not presented in the test trials. After the practice trials, all the participants performed the experiment for a total of 24 trials while the performance was recorded by the examiner sitting on the other side of the room or remotely.

To suppress verbal rehearsal, every trial commenced with two randomly generated digits (from 1 to 9) presented for 1,500 ms and participants were asked to verbally repeat these numbers throughout the whole trial. This articulatory suppression procedure served to prevent participants from verbally rehearsing the names of the shapes and the colours in the study array of six objects, which appeared on the screen for 753 ms. The location of the six items was randomised for each test trial.^1^ The visual array was then followed by a blank screen with a fixation point for 2,000 ms. Following this, participants were asked to orally recall, in any order they chose (verbal free recall) all the combinations of colour–shape bindings shown in the study array. The choice of a 2,000 ms study-test interval followed a previous study by Logie et al. (2011), which showed that for a study-test delay of 1,500 ms or more, randomly changing locations of colour–shape conjunctions between study and test arrays did not disrupt memory performance any more than retaining the same locations for that same interval, or longer, between study and test. In that earlier study, a 2,000 ms study-test interval resulted in performance below ceiling but above floor. In the current experiment, performance using these procedures was below ceiling and well above chance across all 24 trials. Even if participants could recall all 6 individual colours and 6 six individual shapes as individual features, there are 15 possible combinations of colour and shape, so a score of 1/15 or 6.67% would indicate chance, or floor level performance.

**Figure 1.**
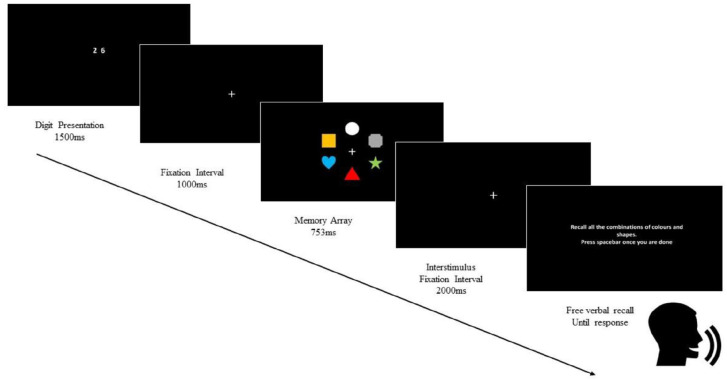
Schematic illustration of the experimental procedure. This procedure was repeated 24 times during the whole learning session.

Numbers of correctly recalled binding combinations for each participant were converted to percentage scores following Shimi and Logie (2019). The minimum score for every trial was 0% (for 0 correct shape–colour combinations recalled out of 6), while the max was 100% (for 6 out of 6 correct shape–colour combinations). Oral responses from participants tested in the lab were recorded by a microphone attached to a pair of headphones while performing verbal free recall of the colour–shape combinations, ignoring location. For participants who were tested remotely via a live video link, no audio recording took place and the participants’ responses were written down on a test sheet by the experimenter.^2^Figure 1 illustrates the experimental procedure.

After the end of the last trial, participants were asked if they became aware of the repetition of the combinations of colours and shapes to assess whether awareness was related to learning of the feature bindings (Shimi & Logie, 2019). Whereas 61 participants reported becoming aware of the repetitions, 59 did not (see Table 1).

Participants were then free to leave the experiment and were told that they would be contacted by phone without specifying the exact day and the reason for the follow-up. Each participant was randomly assigned to one of the three delay conditions for follow-up.

On the phone follow-up, which occurred 1 day or 1 week or 1 month after the learning session, all participants were asked to perform a free verbal recall of the shape–colour combinations that they had seen during the first session. Their response was transcribed, and one point was awarded for each shape–colour combination recalled correctly. Errors were scored either as miss-bindings (e.g., blue triangle instead of the blue circle presented in the learning session), or as omissions (fewer than six items recalled), or as intrusions (recall of a colour or shape that was not in the study array). At free recall, participants were reminded that there were six colour–shape combinations in the array, and they were not limited in their responses and number of attempts (which were not recorded).

Finally, participants were asked whether they tried to actively verbally recall or thought about the bindings between the two testing sessions. At the end of the follow-up session, participants were debriefed about the experiment.

As a follow-up to the question on whether participants became aware or not of the repetition, the additional between-subjects variable of Awareness (Aware vs Unaware) was added for a 3 × 2 × 2 mixed-factorial design, as previously done by Shimi and Logie (2019).

Statistical analysis was computed with R (version 4.0.3). Following the approach used in previous studies (Logie et al., 2009; Shimi & Logie, 2019), responses across the 24 trials were grouped into 4 blocks, each of 6 trials. Previously published studies in the field (Logie et al., 2009; Shimi & Logie, 2019) have presented data from blocks of trials so to reduce noise in the data due to random variation from trial to trial.

Analysis of variance (ANOVA) was used to test whether learning of feature binding had occurred across trial blocks, and to investigate forgetting in the delay between the last block of repeated trials and each of the three delay conditions (1 day, 1 week, and 1 month), separately for participants who reported being aware or being unaware of the repetition. Multiple pair-wise comparisons were computed with a Bonferroni correction using the emmeans package.^3^

## Results

### Online versus onsite testing and effect of array repetition

Before proceeding with formal data analysis, the two subsamples of participants tested onsite and online (60 participants for each group for a total of 120) were compared to verify whether there were any differences in their performance and in the effects of array repetition.

Statistical analysis was performed with a mixed-design ANOVA, with testing venue (online and onsite) as a between-subjects variable and four learning blocks, each comprising six trials (block 1, block 2, block 3, and block 4), as within-subjects variable. Average percentage memory score per block was the outcome variable.

Analysis of variance revealed a non-significant main effect for testing venue, *F*(1,118) = 1.92, *p* = .16, $\eta_{p}^{2}$ = .01, meaning that average percentage memory scores of participants tested online (*M* = 46.88, *SD* = 18.78) or onsite (*M* = 43.46, *SD* = 16.56) were not significantly different.

A significant main effect of learning block was found, *F*(3,354) = 77.65, *p* < .001, $\eta_{p}^{2}$ = .39, so average percentage memory scores increased throughout the four learning blocks (block 1 *M* = 35.21, *SD* = 9.26; block 2 *M* = 42.15, *SD* = 13.53; block 3 *M* = 48.68, *SD* = 18.40; block 4 *M* = 54.62, *SD* = 21.36).

The interaction between testing venue and learning block was not significant, *F*(3,354) = 0.69, *p* = .558, $\eta_{p}^{2}$ = .005, meaning that average percentage memory scores between participants tested online and onsite did not significantly differ at any learning block.

As the average memory performance of participants tested online and onsite did not differ significantly at any stage of the learning procedure, the scores from the two subsamples were merged into a single dataset for remaining analyses.

### Does awareness of repetition influence learning of feature bindings across array repetitions?

**Figure 2.**
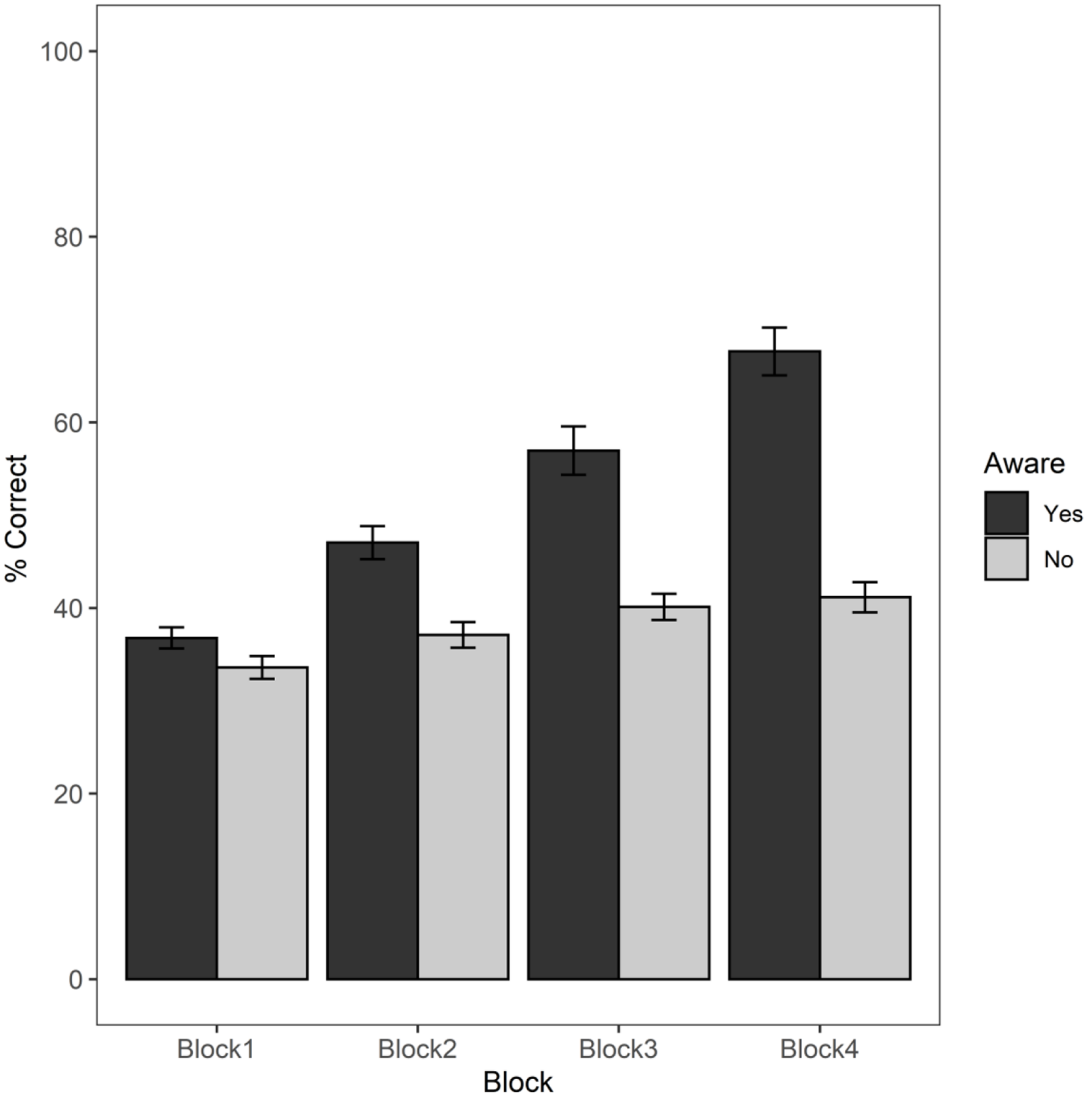
Mean percentage correct recall scores and standard errors for participants reporting being Aware or Unaware of array repetition across four blocks of six trials.

Almost exactly half of the participants (*n* = 61) reported having become aware of the repetition of the colour–shape combinations during the learning procedure, while the other half (n = 59) did not, as shown in Table 1. Mean performance across the four blocks of trials is illustrated in Figure 2, separately for participants reporting being aware or not aware of the repetition.

To analyse these data, a mixed-factorial ANOVA was employed, with level of awareness (aware, unaware) as between-subjects variable, learning block (block 1, block 2, block 3, and block 4) as within-subjects variable and average percentage memory score as outcome variable.

A significant main effect for level of awareness was found, *F*(1,118) = 44.27, *p* < .001, $\eta_{p}^{2}$ = .27, meaning that participants who claimed to have become aware of the repetition pattern generally showed higher average percentage memory scores (*M* = 52.11, *SD* = 20.03) compared with those participants who did not become aware of repetition (*M* = 37.99, *SD* = 11.26).

Also, a significant main effect for learning block was observed, *F*(3,354) = 98.49, *p* < .001, $\eta_{p}^{2}$ = .45, so memory scores significantly improved across learning blocks.

The interaction between level of awareness and learning block was significant, *F*(3,354) = 35.58, *p* < .001, $\eta_{p}^{2}$ = .23. Pair-wise comparisons revealed that the interaction was driven by the consistent differences in performance across repetition blocks 2, 3, and 4 between aware and unaware participants. While memory scores between aware (*M* = 36.77, *SD* = 8.81) and unaware (*M* = 33.59, *SD* = 9.50) participants did not differ at block 1, *t*(236) = –1.24, *p* = .21, meaning that both groups started with the same level of performance, unaware participants achieved lower memory scores at block 2, *t*(236) = –3.87, *p* < .0001 (*M* = 37.09, *SD* = 10.67), at block 3, *t*(236) = –6.55, *p* < .0001 (*M* = 40.12, *SD* = 10.81), and at block 4, *t*(236) = –10.32, *p* < .0001 (*M* = 41.15, *SD* = 12.53), when compared with aware participants at block 2 (*M* = 47.04, *SD* = 13.93), block 3 (*M* = 56.95, *SD* = 20.42), and block 4 (*M* = 67.64, *SD* = 20.06).

Although both groups of participants showed a significant increase in performance across blocks, those participants who eventually became aware of the repetition learned more rapidly and had significantly higher learning rates than those who did not. Notably, unaware participants showed only a 7% increase across trials (34%–41%), even if this was statistically significant. Although aware participants showed a 31% increase from 37% at block 1, they were still well below ceiling (68%) after 24 repetition trials.

### Is there any memory trace after a delay, and is forgetting influenced by whether or not participants were aware of array repetition?

Of the original 120 participants in the first test session, two were not available for retesting at follow-up. Therefore, analyses on forgetting were conducted on the 118 participants tested at follow-up, 40 of which were tested after 1 day, 40 after 1 week, and 38 after 1 month.

At the appropriate time, participants were contacted by phone and were asked to freely recall all the combinations of shapes and colours from the first test session. At follow-up, only 15 participants reported having recalled the bindings during the delay period, while the remaining 103 reported that they had not actively recalled the bindings since the initial test session. A formal analysis of the 15 participants who reported active rehearsal would not be feasible, given that there were too few participants in each group to allow for a meaningful analysis (6 at 1 day, 7 at 1 week, and 2 at 1 month).

To clarify the role of awareness of repetition on long-term retention or forgetting of feature bindings, we included in the analysis whether participants reported being aware or unaware of array repetition. As shown in Table 1, the split between aware and unaware participants during the learning session was almost equal across follow-up conditions (18 aware vs 22 unaware in the 1-day group; 19 aware vs 21 unaware in the 1-week group; 22 aware and 16 unaware in the 1-month group).

To check that the three delay groups did not differ in their level of performance at block 4, before delayed recall was tested, with one-way between participants ANOVAs, we compared the three delay groups on their block 4 performance when any learning would have been maximised for all groups, separately for the aware and unaware participants. This showed that there was no difference at block 4 when comparing the three delay groups for aware participants, *F*(2,56) = 1.73, *p* = .18, $\eta_{p}^{2}$ = .05, or when comparing the groups for unaware participants, *F*(2,56) = 1.77, *p* = .17, $\eta_{p}^{2}$ = .05.

**Figure 3.**
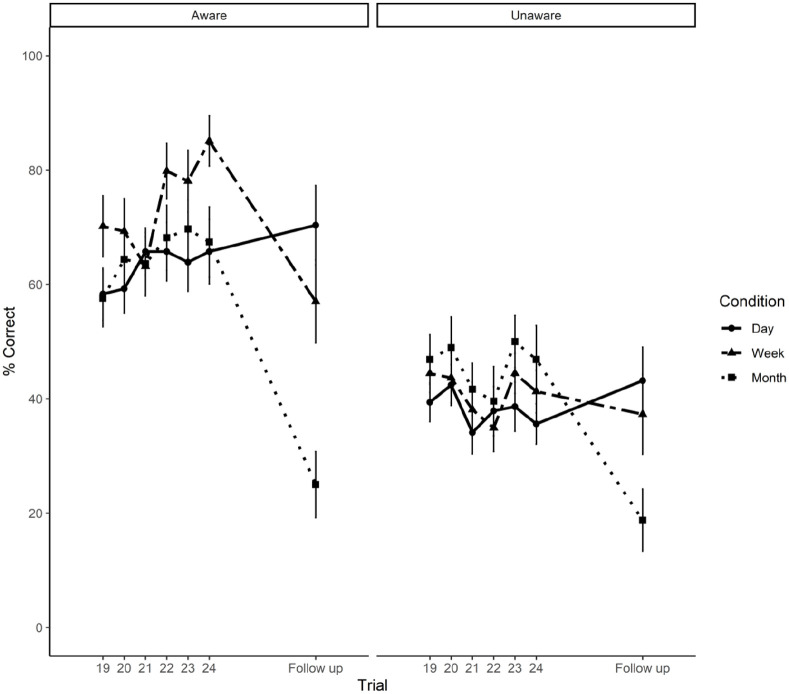
Mean percentage correct recall scores and standard errors from block 4 (19–24) of the learning session to the follow-up session of Aware and Unaware participants assigned to 1 day, 1 week, and 1 month time interval conditions.

We then used a mixed ANOVA to analyse the mean correct recall data comparing block 4 with delayed recall performance across time delay groups (1 day, 1 week, and 1 month) and level of awareness group (aware, unaware). Figure 3 illustrates the mean data for the three delay groups, split by aware and unaware groups, across the six trials of block 4 and for each delay group, and for each delay period.

A significant main effect was observed between the three follow-up groups, *F*(2,112) = 8.21, *p* < .001, $\eta_{p}^{2}$ = .12, and across time delays, *F*(1,112) = 19.32, *p* < .001, $\eta_{p}^{2}$ = .14. There was a significant main effect of level of awareness, *F*(1,112) = 43.71, *p* < .001, $\eta_{p}^{2}$ = .28, as aware participants (*M* = 58.43, *SD* = 29.64) showed higher memory performance compared with unaware participants (*M* = 37.95, *SD* = 22.52).

The interaction between time delay and follow-up condition was significant, *F*(2,112) = 16.26, *p* < .001, $\eta_{p}^{2}$ = .22. The interaction between level of awareness and time delay was not significant, *F*(1,112) = 2.15, *p* = .14, $\eta_{p}^{2}$ = .01. Similarly, the three-way interaction between level of awareness, follow-up condition, and time delay was not significant, *F*(2,112) = 0.85, *p* = .42, $\eta_{p}^{2}$ = .01.

Pair-wise post hoc tests with Bonferroni correction to further explore the significant interaction between time delay and follow-up condition revealed that, while overall memory performance in those participants assigned to the 1-day follow-up condition did not significantly differ from block 4, *t*(112) = –1.29, *p* = .19, memory for feature binding was significantly lower in participants followed up after 1 week, *t*(112) = 2.10, *p* = .03, and after 1 month, *t*(112) = 6.61, *p* < .0001, when compared with block 4.

Therefore, there was no evidence of forgetting after 1 day for aware or unaware participants, but both show significant forgetting after 1 week and 1 month, with the latter showing most forgetting.

Most errors comprised miss-binding of colour and shape from the presented array or omissions. There were only two intrusion errors for which a colour was reported that was not originally displayed (“orange” and “purple”) and only one error for which a shape was recalled that was not initially shown (“box”).

**Figure 4.**
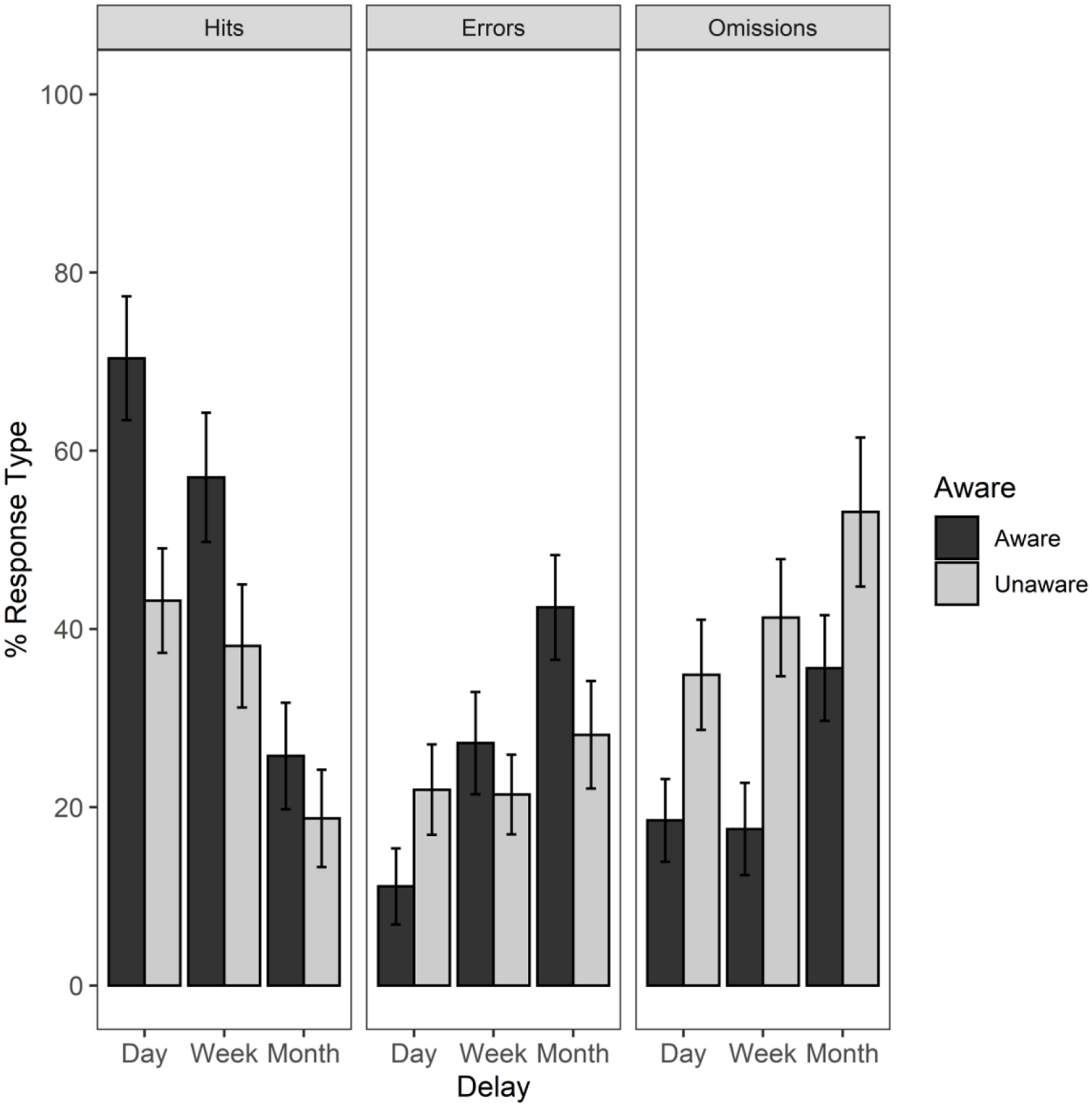
Mean percentage of correct recall scores (Hits), miss-binding errors (Errors), and failures to recall (Omissions) with standard errors, for Aware and Unaware participants assigned to 1 day, 1 week, and 1 month time interval conditions. In this figure, three intrusions were not included in the mean scores.

The errors were then split according to awareness of repetition, as illustrated in Figure 4. The numbers of each type of error were too small for a formal analysis between error types, but from the figure, it is clear that for the 1-month group, errors for aware participants tended to be miss-binding errors, suggesting that they could recall the individual features, but had forgotten most, but not all of the combinations. For unaware participants in the 1-month group, errors tended to comprise omissions, suggesting they could not recall some of the individual features, but did recall a small number of combinations of features that they could remember.

Although correct recall scores after 1 week and 1 month relative to block 4 performance were substantially reduced, even for participants tested after 1 month, performance was around 25% for aware and around 20% for unaware participants. Both scores are well above chance (6.7%). Hence, there was substantial forgetting after 1 month, but there was clear evidence that participants retained some of the material with which they had been presented and recalled 24 times in the initial test session. That is, there is clear evidence that some learning had occurred during the first session, even for the unaware participants.

### How did participants perform before learning occurred compared to how they performed after a delay (comparison between trial 1 vs delayed performance)?

Block 4 was initially considered as the most reliable measure of learning of feature bindings, but this measure derives from the average performance of six learning trials (from 19th until the 24th trial), whereas performance at a follow-up delay was obtained by one, single delayed free recall trial.

Furthermore, block 4 performance resulted after exposure to 24 trials of the same repeated array and therefore represents a measure of episodic long-term memory. Likewise, level of performance in follow-up conditions represents a declining residual trace in long-term episodic memory built upon performance at block 4. A reliable measure of visual short-term memory can be found, instead, through a temporary representation of the items in the array, when participants did not have the chance to learn them, specifically in trial 1 before any repetition has occurred.

To compare how participants performed between delayed recall and how well they performed after only seeing the array once (i.e., with just one presentation), statistical analyses were run by including the average performance on a single trial with respectively no learning in place (trial 1) compared with delayed performance (follow-up interval) on the full sample of those participants who were tested at follow-up (*n* = 118; see Table 1).

**Figure 5.**
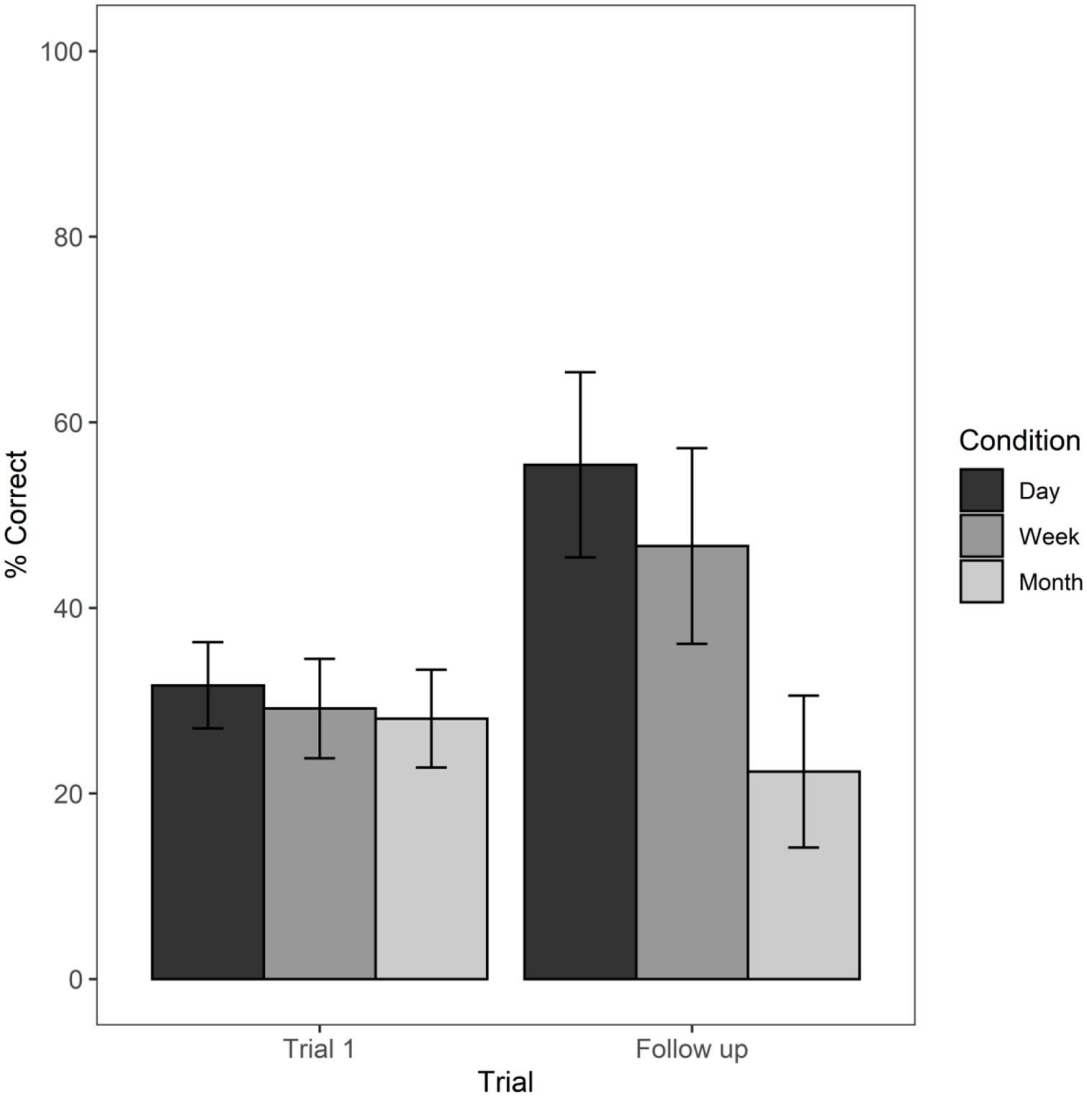
Mean percentage correct recall scores from trial 1 of the learning session to the follow-up assigned to 1 day, 1 week, and 1 month time interval conditions with standard error bars.

Data were analysed with a mixed-factorial ANOVA, with follow-up condition (1 day vs 1 week vs 1 month) as between-subjects variable, time delay as within-subjects variable (trial 1 vs follow-up), and correct recall scores as outcome measure. Mean correct recall scores are illustrated in Figure 5.

Analysis of variance yielded a significant main effect of follow-up condition, *F*(2,115) = 12.51, *p* < .001, $\eta_{p}^{2}$ = .17, a significant main effect of time delay, *F*(1,115) = 13.75, *p* < .001, $\eta_{p}^{2}$ = .10, and a significant interaction between follow-up condition and time delay, *F*(2,115) = 7.74, *p* < .001, $\eta_{p}^{2}$ = .11.

Post hoc pair-wise comparisons with Bonferroni correction revealed that memory performance significantly differed from trial 1 (*M* = 31.66, *SD* = 14.52) to follow-up (*M* = 55.41, *SD* = 31.21) in the 1-day group, *t*(115) = 4.33, *p* < .0001, and from trial 1 (*M* = 26.16, *SD* = 16.77) to follow-up (*M* = 46.66, *SD* = 32.94) in the 1-week group, *t*(115) = 3.19, *p* < .01. Instead, mean memory performance for participants followed up after a 1-month delay (*M* = 22.36, *SD* = 24.89) did not significantly differ from trial 1 (*M* = 28.06, *SD* = 16.02), *t*(115) = −1.01, *p* = .31. Therefore, level of performance after 1 month was approximately the same as on trial 1 of the learning session. However, on trial 1, no learning could have occurred, and, consistent with Shimi and Logie (2019), we would argue that trial 1 performance reflects a capacity limit for a temporary visual memory, or visual cache, whereas performance after 1 month reflects what has been retained in episodic long-term memory after forgetting has occurred.

## Discussion

This study aimed to assess learning of feature bindings by delayed testing after 24 trials of verbal free recall of the same repeated array, as well as patterns of forgetting over long-term intervals (1 day, 1 week, and 1 month), with and without reported awareness of repetition.

### Learning of feature bindings

Memory for feature binding overall improved across trials in which the same combinations of colour and shape were presented repeatedly and tested with verbal free recall. Participants who reported that they had become aware of the repetitions showed markedly faster learning and higher levels of recall of colour–shape conjunctions compared with those participants who reported that they had not been aware of the repetition, although even aware participants did not reach ceiling performance, achieving around 70% correct after 24 repetitions, from a starting baseline of 37%. Although unaware participants showed a statistically significant improvement in recall across trials, this comprised an increase from a baseline of 34% only to around 41% recall performance after 24 repetitions. This extends to a test of verbal free recall, the Logie et al. (2009) observation of no learning across 60 repeated presentations for change detection. The current study also offers a conceptual replication and extension of the findings in Shimi and Logie (2019) Experiment 1 which showed very slow learning with change detection of the same six-item stimulus array repeated for 120 trials, and particularly slow learning in participants who reported being unaware of the repetition.

The slow improvement observed in verbal free recall of a repeated array of colour–shape bindings contrasts with previous studies showing rapid learning over very few trials comprising serial ordered recall of a repeated stimulus (e.g., Couture & Tremblay, 2006; Cunningham et al., 1984; Fastame et al., 2005; Hebb, 1961; Page et al., 2006; Szmalec et al., 2009). It was argued in the Logie et al. (2009), and Shimi and Logie (2019) change detection experiments, that during the first 40–60 repetition trials, participants relied primarily on a visual cache memory, the contents of which were overwritten by the stimulus array on the next trial, even when that array was identical on every trial. There was therefore no residual trace in the visual cache from trial to trial that could support learning. The current study is consistent with that interpretation for the unaware participants, whose performance, even after 24 repetitions with verbal free recall (41% or 2–3 items from the six-item arrays) remained well within the capacity estimate of around 3–4 items for visual short-term memory reported in previous studies (Cowan, 2001, 2005; Luck & Vogel, 1997; Rouder et al., 2008; Vogel et al., 2001). This could be due to the use of verbal free recall of the colour–name pairs causing output interference in contrast with change detection or cued recall that have been used in some previous studies. Accordingly, those studies that previously used verbal free recall for visual arrays (Della Sala et al., 2012; Killin et al., 2018; Parra et al., 2009, 2015) already noted that free recall poses a greater memory load as compared with other tasks involving cued retrieval or a spatial cue.

Moreover, the use of articulatory suppression would have disrupted the use of phonological codes to remember colour or shape names as a supplement to a temporary memory for the visual appearance of the coloured shape. Because Shimi and Logie (2019) found learning across 120 repetition trials, they concluded that, in addition to reliance on a limited, temporary visual cache, there was a weak residual trace in episodic memory that gradually strengthened across trials but required in excess of 40 trials of change detection before that episodic trace was sufficiently strong to support learning and improved memory performance for the repeated array. Only in some participants did the episodic trace become sufficiently strong for them to become aware of the repetition. After 120 repetitions, participants who became aware of the repetition could recall 5–6 items from the six-item array suggesting that, as the number of trials increased, participants gradually relied more on learning in episodic memory and gradually relied less on the limited amount of information that could be held in the visual cache. In the Logie et al. (2009) study, where no learning was observed, we assume that 60 repeated trials of change detection was insufficient for any gradual strengthening of the episodic trace to influence performance.

In sum, our data pattern is consistent with that found in previous studies that have investigated the impact on immediate memory of repeating a visual array. As such, the data can be interpreted as suggesting that during the first few trials in the initial test session, all participants relied on the limited capacity visual cache to support verbal free recall. Over the course of 24 trials with array repetition and verbal free recall, there was a build-up of a weak residual trace in episodic memory, and there was evidence for this in participants who reported becoming aware of the repetition, but not in those who reported being unaware.

One possible account for the latter is that unaware participants continued across all repetition trials to rely primarily on a visual cache with the contents erased and replaced by the array on the next trial, even if identical. Consistent with this interpretation, previous studies have suggested that the capacity limits of visual short-term memory (3–4 items) are likely to be exceeded by the number of items or features in arrays of six or more items (Alvarez & Cavanagh, 2004). Moreover, bindings in visual short-term memory have been shown to be fragile and susceptible to interference on a trial-by-trial basis (Allen et al., 2006; Baddeley et al., 2011). However, it was unclear from those previous studies, from the Logie et al. (2009) and Shimi and Logie (2019) studies, and on first analysis, it was unclear from our current data, that if participants show no evidence of learning across trials, they were actually learning only a subset of the six-item array, and so showed little or no improvement across 24 repetitions. This was addressed in the current study by investigating recall performance after delays of 1 day, 1 week, and 1 month.

### Delayed verbal free recall

Overall, our data for delayed recall indicate that memory for feature bindings decreases only after longer time intervals of 1 week or 1 month, while it seemed to remain stable after an interval of 1 day regardless of the amount of learning that participants achieved after 24 trials. Thus, the episodic memory trace for learned colour–shape bindings appeared to remain stable up to 24 hr after the last block of the learning session for both aware and unaware participants. Because participants were not presented with the array after the initial test session, the lack of any evidence for forgetting after 1 day, and limited forgetting after 1 week offers strong evidence for learning during repetition, even among the participants who reported being unaware of the repetition at the end of the initial test session. Although performance was even lower for the group tested after 1 month, indicating further forgetting, even for the unaware participants in this group, performance was still well above chance performance.

Further analyses compared how participants performed between delayed recall and how well they performed after only seeing the array once (trial 1). Performance after 1 month was the same as on trial 1. However, no learning could have occurred on trial 1, but if performance after 24 repetitions was based on no learning as on trial 1, we would have expected chance performance with delayed recall after 1 day, which was clearly not the case. Nor was it the case even after a delay of 1 month. So this evidence suggests that even the participants who showed little or no improvement across the 24 repetitions had learned at least part of the array and retained some of that learning for up to 1 month. These results are consistent with the hypothesis that two cognitive functions support memory performance: a limited capacity visual cache supports performance in early trials, and a strengthening episodic trace increasingly supports performance as learning progresses. Any lack of performance improvement across trials can be interpreted as learning of a subset of the six-item array some of which can be recalled up to 1 month later.

When considering the factor of awareness of repetition, aware participants were able to correctly recall more items from the array than unaware participants. These results reiterate the role of awareness of repetition in long-term learning for feature binding. Among aware participants, the additional component of long-term episodic memory during learning contributed to the generation of a stronger memory trace (Shimi & Logie, 2019). One possible account is that as these participants became aware of the repetition, they attempted to learn additional items in the array beyond those that could be retained in a temporary visual cache. In contrast, participants who did not become aware of the repetition may have continued to focus on just a subset of the array that could be held in visual short-term memory, but with a strengthening episodic trace across trials for only that subset of items, hence showing no improvements in performance across repetitions. However, the strengthened episodic trace for a subset of the array was sufficient to support recall after delays of up to 1 month.

### A possible caveat and conclusions

Asking participants at the end of the repetition trials, whether they were aware of the array repetition yielded new findings regarding the process of learning, and the role of awareness of repetition of a visual array for verbal free recall. A possible caveat is that asking about repetition awareness might have biased subsequent delayed recall performance, e.g., by prompting spontaneous rehearsal of shape–colour combinations during the 24 hr after the initial test session. This might account for the lack of forgetting when testing the recall after 1 day and could account for the retention of some of the colour–shape bindings after 1 week and 1 month. However, only a small number of participants, when asked, reported that they had actively thought about the items after the initial test sessions, so this is very unlikely to have resulted in an over-estimation of recall performance across all participants and across all three delay periods.

By exploring retention after extended delays, this study also revealed that learning of at least a subset of a repeated array can occur, even when participants are unaware of the repetition, and, crucially, when they show no evidence of performance improvement across repetitions. Moreover, the study showed how learned feature bindings are retained at long-term intervals. Whereas such learned bindings could be retained up to 1 day, recall declined after periods of 1 week and 1 month, regardless the level of awareness of the initial repetition. However, even after a month, there remained memory for some of the learned feature bindings, even for participants who were unaware of the repetition and showed no evidence of learning during the repeated trials.

Taken together, these findings provide a deeper insight into the impact of array repetition on retention of feature bindings. Long-term learning does occur across repetitions, even if participants are unaware of the repetition and show little or no evidence of learning during the repetition trials. The evidence is consistent with performance being supported by both a temporary visual cache, sometimes also referred to as visual short-term memory, and by gradually increasing support from a strengthening episodic trace in long-term memory.

## Notes

1 Although previous reports highlighted the central role of location in binding (see Treisman & Zhang, 2006), we ran a pilot study with items in fixed locations across trials and performance was close to ceiling after 24 repetitions. Randomising the locations of the items in the array for each trial while maintaining the same colour–shape combinations resulted in scores below ceiling, but well above floor.

2 The ethical approval required participants to physically sign a consent form to allow for audio recording that took place in the lab, and this was impractical with remote testing via a live video link.

3 Statistical analyses were also performed by using linear mixed effects modelling as implemented by package lmer4 (Bates et al., 2015) in R. These models also included a random intercept for participants (1|ID) and they yielded the same results as with analyses of variance (ANOVAs).
